# Identification of Novel piR-2158 Isoforms and Their Distinct Antitumor Effects on Triple-Negative Breast Cancer

**DOI:** 10.3390/cancers18142237

**Published:** 2026-07-12

**Authors:** Zhongrui Wang, Yu Liu, Lu Qian, Jiayuan Li, Zuoren Yu, Qian Zhao, Jinhui Lü

**Affiliations:** 1 Medical Innovation Center and Research Center for Translational Medicine, Shanghai East Hospital, Tongji University School of Medicine, 150 Jimo Road, Shanghai 200120, China; wangzr2001@126.com (Z.W.); liu_yu_96@163.com (Y.L.); ql1206733047@126.com (L.Q.); lmz10071131@outlook.com (J.L.); zuoren.yu@tongji.edu.cn (Z.Y.); 2 Department of General Surgery, Shanghai East Hospital, Tongji University School of Medicine, Shanghai 200120, China; 3 Department of Ultrasound Medicine, Shanghai East Hospital, Tongji University School of Medicine, Shanghai 200120, China; 4 Department of Laboratory Medicine, Shanghai East Hospital, Tongji University School of Medicine, Shanghai 200120, China

**Keywords:** breast cancer, piRNA, piR-2158, isoform

## Abstract

Background: The diversity of RNA isoforms plays a critical role in regulating the development and progression of human cancers. Our previous work has demonstrated that piR-2158 exerts antitumor activity in breast cancer by repressing IL11-STAT3 signaling. Methods: The isoforms of piR-2158 were analyzed using Sanger sequencing, quantitative real-time PCR (qRT-PCR), and Gene Expression Omnibus (GEO) dataset. Cell proliferation capacity was assessed using Cell Counting Kit-8 (CCK-8) assays and Ki67 immunofluorescence staining. Cell migration was evaluated using wound healing assays. Cancer cell stemness was analyzed using mammosphere formation assay, stemness marker detection, and ALDH activity assay. Results: We identified two types of piR-2158 isoforms in mammary tissues: a 31 nt long isopiR (designated as iso-piR-2158-L) and a 28 nt short isopiR (designated as iso-piR-2158-S). Predominant expression of iso-piR-2158-L was observed in normal mammary epithelial cells and adjacent non-tumor breast tissues, whereas it was significantly downregulated in TNBC cell lines and primary tumor tissues. We experimentally demonstrated that iso-piR-2158-L and iso-piR-2158-S exert distinct effects on TNBC cell proliferation, migration, and stemness, with iso-piR-2158-L showing significantly stronger antitumor effects than iso-piR-2158-S. Subsequent mechanistic studies revealed that iso-piR-2158-L suppresses IL11 expression more effectively, compared to iso-piR-2158-S. Conclusions: Our findings reveal a piRNA isoform-based regulatory pathway that may be involved in regulating pathological transformation, tumor initiation, and progression in TNBC.

## Simple Summary

The expression profiles and functional roles of piRNA isoforms (isopiRs) in human malignancies remain poorly characterized. In the current study, we identified two novel piR-2158 isoforms in human breast tissues and cell lines: a 31-nucleotide (nt) isoform (iso-piR-2158-L) and a 28 nt isoform (iso-piR-2158-S). Iso-piR-2158-L is the predominantly expressed isoform in normal mammary tissue, whereas its expression is significantly downregulated in triple-negative breast cancer (TNBC), functioning as a tumor suppressor by inhibiting breast cancer cell proliferation, migration, and stem-like properties. In addition, iso-piR-2158-L exhibits greater suppressive efficacy against IL11 expression than iso-piR-2158-S. Collectively, these findings uncover a previously uncharacterized piRNA isoform-based regulatory pathway in TNBC.

## 1. Introduction

Piwi-interacting RNAs (piRNAs) are a class of small non-coding RNAs (ncRNAs) approximately 24–31 nucleotides (nt) in length, predominantly expressed in germ cells [1,2]. By binding to PIWI proteins, piRNAs assemble into ribonucleoprotein complexes that mediate transposon silencing, epigenetic regulation, and maintenance of genomic stability [1,2]. Emerging evidence has revealed aberrant expression of certain piRNAs in multiple types of cancer cells [3,4], especially in cancer stem cells (CSCs) [5]. These piRNAs have been reported to be involved in the regulation of tumor initiation and progression, or to serve as potential biomarkers for cancer diagnosis and prognosis [6,7,8].

RNA isoforms are functionally diverse RNA variants generated from the same gene locus or same precursor RNA transcript via multiple post-transcriptional processing mechanisms, including alternative splicing, shifted endonucleolytic cleavage sites, or differential methylation modifications. The diversity of RNA isoforms plays a critical role in regulating the development and progression of human cancers [9,10,11]. RNA isoforms have been identified in both protein-coding mRNAs and non-coding RNAs. For instance, several genes have been found to exhibit varying lengths of 3′ untranslated regions (3′UTRs) between the breast cancer cell lines MCF-7 and MDA-MB-231 [12]. Non-coding microRNA (miRNA) isoforms, termed isomiRs, were first reported in 2008 [13]. IsomiRs differ from canonical reference miRNA sequences by one or more nucleotides, driven by variable Drosha/Dicer cleavage positions and/or non-templated nucleotide additions [14,15]. Similarly, piRNAs also produce distinct isoform variants, designated as isopiRs. However, the understanding of isopiRs remains far less comprehensive than that of isomiRs.

piR-2158, also named DQ572892, piR-2980, piRNA-21067, piR-41004, piR-3200, and piR-34003 by different databases, is located on the reverse strand of chromosome 16 with 29 nt in length [16,17]. piR-2158 is expressed across germ cells [18], mesenchymal stromal cells (MSCs) [19], and cancer cells [16,17]. Our previous work has demonstrated downregulation of piR-2158 in breast cancer stem cells [17]. Both in vitro and in vivo assays have demonstrated that piR-2158 exerts robust antitumor activity in breast cancer by competitively binding to the *IL11* promoter region alongside the transcription factor FOSL1, thereby repressing IL11-STAT3 signaling [17].

In the current study, we identified two types of novel piR-2158 isoforms in normal and tumor mammary tissues, as well as cell lines. Further analysis of the 31 nt long isopiR (iso-piR-2158-L) and 28 nt short isopiR (iso-piR-2158-S) demonstrated dominant expression of iso-piR-2158-L in normal mammary epithelial cells and adjacent non-tumor breast tissues, whereas there was a significant downregulation in TNBC cell lines and primary tumor tissues. In addition, we experimentally demonstrated that iso-piR-2158-L exerts stronger antitumor effects on TNBC cell proliferation, migration, and stemness, compared to iso-piR-2158-S. A mechanistic study found that iso-piR-2158-L has a higher efficacy than iso-piR-2158-S for suppressing IL11 expression. These findings reveal a piRNA isoform-based regulatory pathway that may regulate pathological transformation and tumor initiation in TNBC.

## 2. Methods and Materials

**Clinical tissue specimens.** Tumor tissues and adjacent normal tissue samples were collected from triple-negative breast cancer patients (*n* = 3, stage III) during surgery at Shanghai East Hospital. Those patients did not receive neoadjuvant therapy before surgery. Adjacent normal tissues were sampled at least 2 cm from the primary tumor margin without pathological abnormalities. All procedures received approval from the Institutional Review Board of Shanghai East Hospital (#2019TJDX107). All patients were provided with written informed consent forms.

**Cell lines and cell culture.** The cell lines used in this study are all commercially available, originally purchased from the American Type Culture Collection (ATCC, Manassas, VA, USA) and the Cellcook Biotech Co., Ltd. (Guangzhou, China), and maintained in our lab. Briefly, the human normal mammary epithelial cell line MCF-10A (ATCC ID: CRL-10317) was cultured in Dulbecco’s Modified Eagle Medium/Nutrient Mixture F12 (DMEM/F12) supplemented with 5% horse serum, 1% penicillin/streptomycin (P/S), 20 ng/mL epidermal growth factor (EGF), 10 μg/mL insulin, 0.5 μg/mL hydrocortisone, and 0.1 μg/mL cholera toxin. Human breast cancer cell lines MDA-MB-231 (ATCC ID: HTB-26), BT549 (ATCC ID: HTB-122), Hs578T (ATCC ID: HTB-126), and SUM159 (Cellcook ID: CC0326) were cultured in DMEM supplemented with 10% fetal bovine serum (FBS) and 1% P/S. All cells were cultured in a 37 °C, 5% CO_2_ incubator. All cell lines are authenticated and receive routine detection of mycoplasma contamination.

**qRT-PCR assay**. Total RNA was extracted using the TRIzol (Invitrogen, Carlsbad, CA, USA) method. To achieve more specific detection of piR-2158 isoforms, we applied the stem-loop-stem method for iso-piR-specific reverse transcription, which can distinguish isoforms with a single base difference. For the subsequent PCR amplification, an iso-piR-specific forward primer was used for each isoform. cDNA of mRNAs was prepared using HiScript III all-in-one RT SuperMix (Vazyme R333, Nanjing, China). All primers were synthesized by Nanjing GenScript Biotechnology Co., Ltd. (Nanjing, China). Quantitative real-time PCR (qRT-PCR) analyses were carried out using SYBR Green PCR Master Mix on a ABI QuantStudio™ 6 Flex systerm (Thermo Fisher Scientific, Singapore, Singapore); 5s rRNA and GAPDH were used for normalization of piRNAs and mRNAs, respectively. The primer sequences are listed in Supplementary Table S1.

**piRNA sequencing analysis.** The PCR products of total piR-2158 were purified using the SanPrep Column PCR Product Purification Kit (B518141-0100, Sangon, Shanghai, China), followed by ligation into the pMD19-T vector and transformation into *E. coli* competent cells. Plasmid DNA was extracted from individual single clones. Two clones were randomly selected from each sample for Sanger sequencing analysis (Beijing Liuhe BGI Co., Ltd., Shanghai, China).

**Oligos and cell transfection.** All piRNA mimic oligos, including the non-targeting negative control (NC) with scrambled sequences, were synthesized by Nanjing GenScript Biotechnology Co., Ltd. (Nanjing, China). Oligos were transfected into cells using Lipofectamine RNAiMAX (Invitrogen 13778150, USA) at a final concentration of 20 nM. The sequence information of all piRNA mimic oligos can be found in Supplementary Table S2.

**CCK-8 assay.** Following, 1 × 10^3^ cells/well were seeded into 96-well plates with 4 replicates per group. After incubation for 0, 24, 48, or 72 h, 10 μL of CCK-8 solution was added to each well, followed by a 3 h incubation in a 37 °C, 5% CO_2_ incubator. Absorbance at 450 nm was measured for quantitative analysis of cell proliferation.

**Ki67 immunofluorescence staining assay.** Cells were seeded onto sterilized coverslips at a 30% density, with 3 replicates per group. After 24 h of incubation, the cells were fixed with 4% paraformaldehyde at room temperature for 15 min, followed by permeabilization with 0.3% Triton X-100, blocking with 1% BSA, and hybridization with anti-Ki67 antibody (1:200 dilution, ABclonal A20018, Wuhan, China) overnight at 4 °C. Subsequently, cells were incubated with a fluorescent secondary antibody (Alexa Fluor^®^ 488, Goat Anti-Rabbit IgG H&L, 1:500 dilution, Abcam ab150077, Cambridge, UK) at room temperature for 1 h. An amount of 10 μg/mL DAPI was used to stain nuclei. Leica DM6000 B microscope (Leica Microsystems, Wetzlar, Germany) was used for photography. Three fields were randomly selected per section for quantitative analysis using ImageJ 1.5.0 (National Institutes of Health, Bethesda, MD, USA). The Ki67 index was calculated as: Ki67+(%) = (number of Ki67-positive nuclei/total number of nuclei counted) × 100%.

**Wound healing Assay.** Following, 2 × 10^5^ cells/well were seeded into a 6-well plate with 3 replicates per group. When the cell density reached 90%, the medium was replaced with DMEM containing 0.1% FBS for 24 h to minimize cell proliferation. A vertical wound was created in each well using a 10 μL pipette tip. Five fields were randomly selected in each well at each time point for microscope photography. Cell migration ability was calculated with relative wound breadth. Relative wound breadth = B_n_/average B_0_, where B_0_ is the initial wound breadth, and B_n_ is the residual wound breadth at the tested time point.

**Mammosphere formation assay.** Following, 2 × 10^3^ cells/well were seeded into a 6-well Ultra-Low Attachment Plate (Corning 3471, Corning, NY, USA) with 3 replicates per group for 7-day suspension culture. The culture medium consisted of DMEM/F12 supplemented with 2% B-27 (Gibco 17504044, Waltham, MA, USA), 20 ng/mL EGF (MACGENE CC102CF, Beijing, China), 20 ng/mL bFGF (Gibco 13256-029, Grand Island, NY, USA), and 5 mg/mL insulin (MACGENE CC101, Beijing, China). Leica microscope was used for photography. Only those spheres with a diameter ≥ 35 μm were counted. Three fields were randomly selected in each well for quantitative analysis using ImageJ.

**Western blot assay.** The following primary antibodies (1:1000 dilution) were used for Western blot: anti-SOX2 (Cell Signaling Technology 3579, Danvers, MA, USA), anti-KLF4 (Arigo ARG-55811, Taiwan, China), anti-NANOG (Cell Signaling Technology 4903S, Danvers, MA, USA), anti-IL11 (Proteintech 55169-1-AP, Wuhan, China), and anti-GAPDH (Santa Cruz sc-47724, Dallas, TX, USA). HRP-linked anti-rabbit IgG (Cell Signaling Technology 7074S, Danvers, MA, USA) and HRP-linked anti-mouse IgG (Cell Signaling Technology 7076S, Danvers, MA, USA) were used as secondary antibodies (1:5000 dilution).

**ALDH Assay.** Aldehyde Dehydrogenase (ALDH) activity in breast cancer cells was assessed using the ALDH Green Fluorescent Staining Kit (Stansform STF18001, Nanjing, China) according to the manufacturer’s instructions. Briefly, trypsin-generated single cells were suspended in ALDH fluorescent reagent-containing buffer, incubated at 37 °C for 30 min with or without the ALDH inhibitor Diethylaminobenzaldehyde (DEAB), and then subjected to FACS analysis on a FACScan flow cytometer (Agilent NovoCyte, Santa Clara, CA, USA). Data processing was completed using FlowJo v10.6.2 (FlowJo, LLC, Ashland, OR, USA).

**GEO public data assay.** Expression of piRNA-2158 variations in clinical samples was further validated using a small RNA-seq dataset and BLASTN (v2.17.0) analysis of read sequences. Raw sequencing data at GSE279780 (23 TNBC tumor tissues and 24 normal mammary control tissues) were downloaded from the Gene Expression Omnibus (GEO) database using the SRA Toolkit prefetch utility. FASTQ files were assessed for sequencing quality using FastQC (v0.11.9), followed by adapter trimming with Trim Galore (v0.6.7). Processed reads were converted from FASTQ to FASTA format using the Bio module in Python 3.12 (Python Software Foundation, Beaverton, OR, USA).

**Statistical analysis.** Data are presented as mean ± SD unless otherwise stated. For two-group comparisons, two-tailed independent-samples *t*-tests were used for normally distributed data, and the nonparametric Mann–Whitney U test was applied when normality was not satisfied. All group comparisons involving three or more groups were performed using one-way analysis of variance (ANOVA). When the data conformed to the assumptions of normality and homogeneity of variances, the post hoc least significant difference (LSD) test was adopted for multiple comparisons; when the homogeneity of variance assumption was violated, Dunnett’s T3 test was applied instead. *p* < 0.05 was defined as statistically significant. All statistical analyses were performed using IBM SPSS Statistics 22.0 (Armonk, NY, USA). Graphs were generated with GraphPad Prism 9.5 (San Diego, CA, USA).

## 3. Results

**Figure 1:**
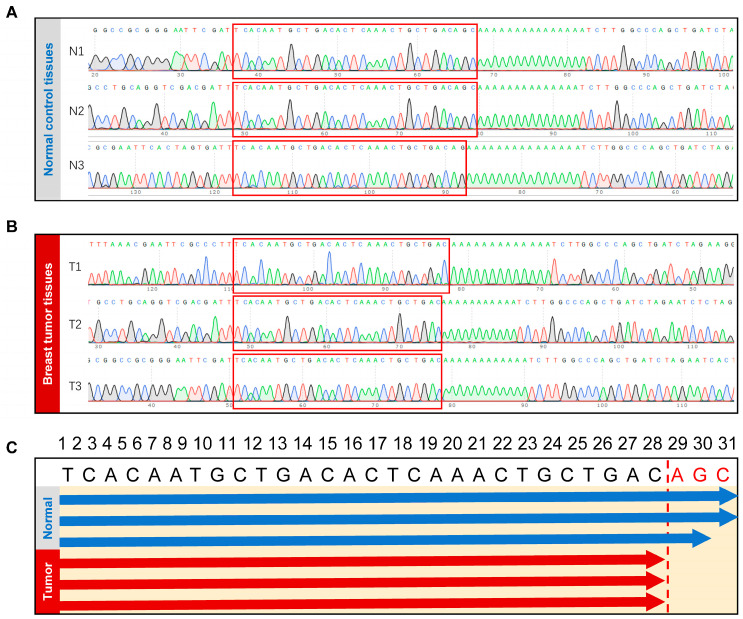
Identification of piR-2158 isoforms in normal mammary epithelium and breast cancer tumor tissues. (**A**,**B**) Representitative raw sequence data of piR-2158 isoforms in normal mammary epithelium (**A**) and breast cancer tumor tissues (**B**) using Sanger sequencing analysis. The rectangles with red line display sequences of the piR-2158 isoforms. (**C**) FASTA format of piR-2158 isoforms in (**A**,**B**), ranging from 28 nt to 31 nt in length. The red dashed line indicates the isoforms with additional nucleotides (nt letters in red) at the 3′ end, compared to the 28-nt short isoform. Two clones were randomly selected from each sample for sequencing. Among the six clones from normal tissue samples, one failed with sequencing, three were identical to the 31 nt isoform, and the remaining two clones were identical to the 30 nt and 28 nt isoforms, respectively. Among the six clones from tumor tissue samples, five were identical to the 28 nt isoform, and one to the 31 nt isoform.

**Identification of piR-2158 isoforms in human mammary epithelium and tumors.** Our previous work has revealed aberrant expression and tumor suppressive function of piR-2158 in breast cancer [17]. To further validate and compare piR-2158 features across normal mammary epithelial cells and breast cancer cells, we amplified piR-2158 from both tumor tissues and adjacent normal tissues collected from breast cancer patients with TNBC, followed by Sanger sequencing analysis. Unexpectedly, we identified distinct piR-2158 isoforms ranging from 28 nt to 31 nt in length. These piR-2158 isoforms share an identical 5′ end sequence but differ at their 3′ ends (Figure 1A–C). Notably, the long isoforms (30–31 nt) were predominantly expressed in normal mammary tissues (Figure 1A,C), while the short isoform (28 nt) was more detected in breast cancer tumor tissues (Figure 1B,C).

**Figure 2:**
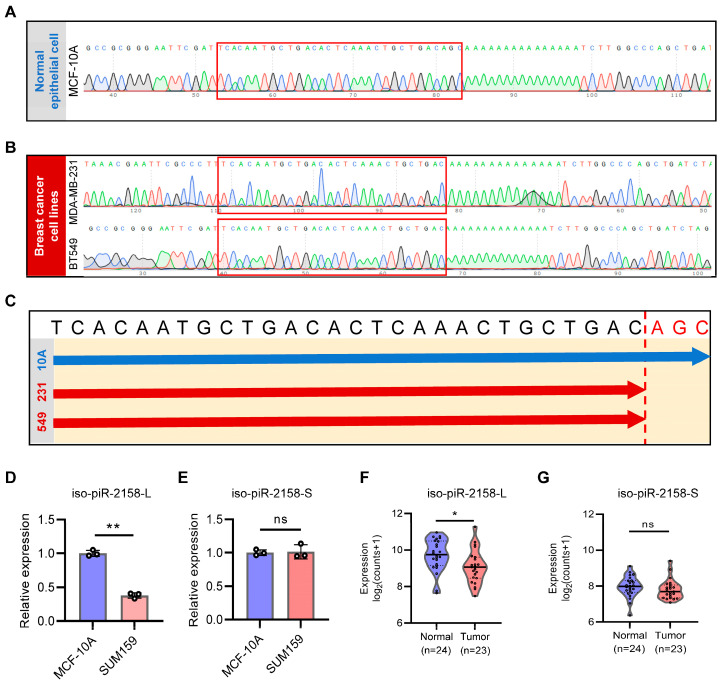
Validation of piR-2158 isoforms in human mammary epithelial cells and breast cancer cell lines. (**A**,**B**) Representitative raw sequence data of piR-2158 isoforms in the normal mammary epithelial cell line MCF-10A (**A**) and two breast cancer cell lines MDA-MB-231 and BT-549 (**B**). The rectangles with red line display sequences of the piR-2158 isoforms. (**C**) FASTA format of piR-2158 isoforms in (**A**,**B**). The red dashed line indicates the isoforms with additional nucleotides (nt letters in red) at the 3′ end, compared to the 28-nt short isoform. Two clones were randomly selected from each sample to apply sequencing. The 31 nt isoform was detected in both of the two clones from MCF-10A, while the 28 nt isoform was detected in three of the four clones from the TNBC cell lines. The remaining clone carried the 30 nt isoform. (**D**,**E**) qRT-PCR analysis of iso-piR-2158-L (**D**) and iso-piR-2158-S (**E**) in TNBC cell line MDA-MB-231 and normal mammary epithelial cell line MCF-10A. Data are presented as mean ± SD (*n* = 3). (**F**,**G**) Gene expression analysis of iso-piR-2158-L (**F**) and iso-piR-2158-S (**G**) in 23 TNBC tumor tissues and 24 normal mammary control tissues using the GSE279780 dataset. Data are presented as median ± quartiles. ns: not significant, * *p* < 0.05, ** *p* < 0.01.

To further validate these findings, we performed similar Sanger sequencing of piR-2158 in the normal mammary epithelial cell line MCF-10A and two TNBC cell lines (MDA-MB-231 and BT-549). As a result, the 31 nt isoform was detected in MCF-10A cells (Figure 2A,C), while the 28 nt isoform was more detected in MDA-MB-231 and BT-549 cells (Figure 2B,C). Based on the sequence length of the dominant piR-2158 isoforms we detected, the 31 nt isoform was designated as iso-piR-2158-L, and the 28 nt isoform as iso-piR-2158-S.

In order to validate the Sanger sequencing results, we performed the stem-loop-stem reverse transcription approach-based qRT-PCR analysis to quantify the abundance of iso-piR-2158-L and iso-piR-2158-S in MCF-10A and TNBC cell line SUM159. As illustrated in Figure 2D, iso-piR-2158-L was confirmed to be the dominant isoform of piR-2158 in MCF-10A, whereas its expression is significantly downregulated in SUM159 cells. Notably, the abundance of iso-piR-2158-S did not show a difference between the two cell lines (Figure 2E). We further analyzed the abundance of iso-piR-2158-L and iso-piR-2158-S in 23 TNBC tumor tissues and 24 normal mammary control tissues using the GSE279780 dataset. Similar to the results in cell lines, iso-piR-2158-L showed higher expression in tumor tissues than normal controls (Figure 2F), while no significant difference was observed for iso-piR-2158-S between the two groups (Figure 2G).

**Figure 3:**
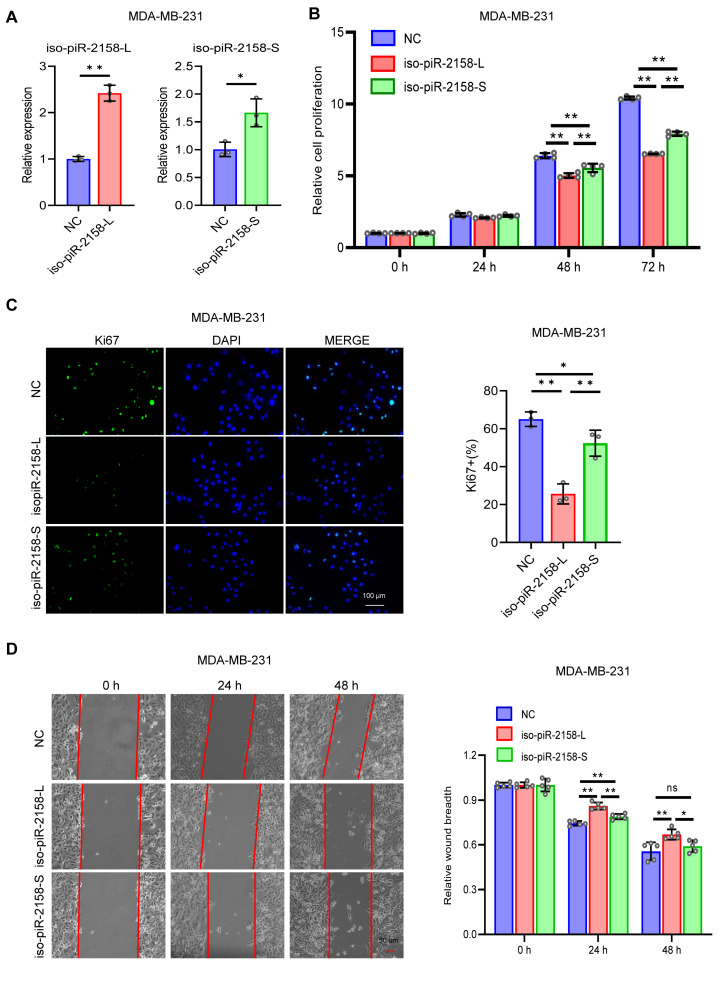
Distinct inhibitory effects of iso-piR-2158-L and iso-piR-2158-S on proliferation and migration of MDA-MB-231 breast cancer cells. (**A**) Validation of the transfection efficiency of iso-piR-2158-L and iso-piR-2158-S in MDA-MB-231 cells. NC: negative control. (**B**,**C**) CCK-8 (**B**) and Ki67 staining (**C**) assays indicating a significant suppression of MDA-MB-231 cell proliferation by both iso-piR-2158-L and iso-piR-2158-S (compared to NC), with iso-piR-2158-L showing a stronger suppressive effect on proliferation than iso-piR-2158-S (iso-piR-2158-L vs. iso-piR-2158-S). (**D**) Wound-healing assay showing a significant suppression of cell migration by iso-piR-2158-L in MDA-MB-231 cells, compared to either NC or iso-piR-2158-S. The red lines indicate the boundaries of the scratches. Data are presented as mean ± SD (*n* = 4 in B, *n* = 5 in D, *n* = 3 in others); ns: not significant, * *p* < 0.05, ** *p* < 0.01.

**Distinct inhibitory effects of iso-piR-2158-L and iso-piR-2158-S on breast cancer cell proliferation and migration.** To determine the biological regulatory roles of iso-piR-2158-L and iso-piR-2158-S in TNBC, we synthesized sequence-specific piRNA mimics for both isoforms, as well as a non-targeting negative control (NC) bearing a scrambled sequence. Following transfection into the TNBC cell line MDA-MB-231 (Figure 3A), CCK-8 cell viability and Ki67 immunostaining assays revealed that both iso-piR-2158-L and iso-piR-2158-S inhibit breast cancer cell proliferation (Figure 3B,C). Notably, iso-piR-2158-L exerted a significantly stronger suppressive effect on proliferation than iso-piR-2158-S (Figure 3B,C). In addition, wound-healing assays demonstrated that iso-piR-2158-L significantly suppressed MDA-MB-231 cell migration, compared to the NC group (iso-piR-2158-L vs. NC, Figure 3D). Furthermore, iso-piR-2158-L showed a significantly stronger suppressive effect on migration than iso-piR-2158-S (iso-piR-2158-L vs. iso-piR-2158-S, Figure 3D).

**Figure 4:**
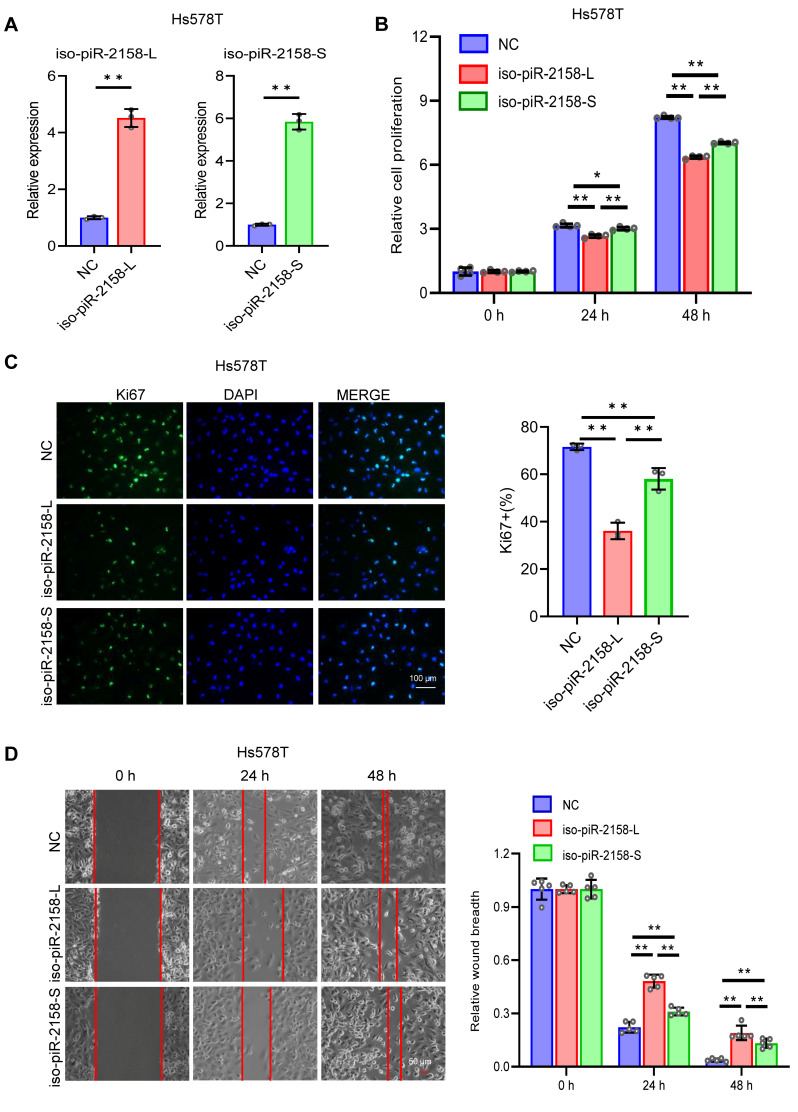
Validation of inhibitory effects of iso-piR-2158-L and iso-piR-2158-S on proliferation and migration of another breast cancer cell line, Hs578T. (**A**) Validation of the transfection efficiency of iso-piR-2158-L and iso-piR-2158-S in Hs578T cells. NC: negative control. (**B**,**C**) CCK-8 (**B**) and Ki67 staining (**C**) assays indicating a significant suppression of cell proliferation by both iso-piR-2158-L and iso-piR-2158-S, with iso-piR-2158-L showing a stronger inhibitory effect on cell proliferation than iso-piR-2158-S. (**D**) Wound-healing assay demonstrating that both iso-piR-2158-L and iso-piR-2158-S significantly suppressed cell migration in Hs578T cells (compared to NC), and iso-piR-2158-L showed a stronger suppressive effect on cell migration than iso-piR-2158-S (iso-piR-2158-L vs. iso-piR-2158-S). The red lines indicate the boundaries of the scratches. Data are presented as mean ± SD (*n* = 4 in B, *n* = 5 in D, *n* = 3 in others). * *p* < 0.05, ** *p* < 0.01.

In order to further validate the distinct tumor-suppressing function of iso-piR-2158-L and iso-piR-2158-S in breast cancer, another breast cancer cell line, Hs578T, was transfected with either iso-piR-2158-L or iso-piR-2158-S, followed by cell proliferation and migration assays. As shown in Figure 4A–D, similar results were obtained in Hs578T as in MDA-MB-231 (Figure 3).

**Figure 5:**
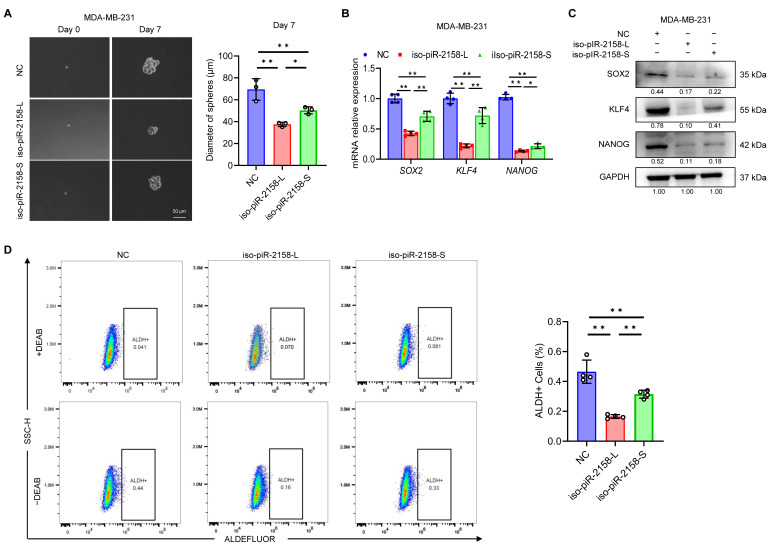
Distinct effects of iso-piR-2158-L and iso-piR-2158-S on suppression of cancer cell stemness in MDA-MB-231 cells. (**A**) In vitro mammosphere formation assays showing that both iso-piR-2158-L and iso-piR-2158-S significantly suppress mammosphere formation, with iso-piR-2158-L exerting a markedly stronger inhibitory effect than iso-piR-2158-S. (**B**,**C**) qRT-PCR and Western blot analyses confirmed that both piR-2158 isoforms downregulate expression of the key stemness markers SOX2, KLF4, and NANOG, with iso-piR-2158-L inducing a significantly greater reduction in marker expression than iso-piR-2158-S. (**D**) Suppression of the ALDH^+^ cancer stem cell population by both iso-piR-2158-L and iso-piR-2158-S (compared to NC), with iso-piR-2158-L showing a stronger CSC-suppressive effect than iso-piR-2158-S (iso-piR-2158-L vs. iso-piR-2158-S). Data are presented as mean ± SD (*n* = 3 in A, *n* = 4 in others). * *p* < 0.05, ** *p* < 0.01. The uncropped blots are shown in Supplementary Materials.

**Distinct effects of iso-piR-2158-L and iso-piR-2158-S on suppression of breast cancer stem cells.** In view of the stem cell regulation of piRNAs, we evaluated the impacts of iso-piR-2158-L and iso-piR-2158-S on breast cancer cell stemness. Upon transfection with mimics of either iso-piR-2158-L or iso-piR-2158-S into MDA-MB-231 cells, both showed significant suppression of mammosphere formation (Figure 5A). However, the size of spheres formed by iso-piR-2158-L-overexpressing cells was significantly smaller than those formed by iso-piR-2158-S-overexpressing cells (Figure 5A). We additionally performed qRT-PCR and Western blot analyses of the cell stemness markers SOX2, KLF4, and NANOG, indicating that both piR-2158 isoforms inhibited the expression of these genes, although a significantly greater reduction was observed with iso-piR-2158-L than with iso-piR-2158-S (Figure 5B,C, Supplementary Table S3). Additionally, ALDH activity assays further confirmed that both iso-piR-2158-L and iso-piR-2158-S significantly suppressed the ALDH^+^ CSC subpopulation relative to the NC group (Figure 5D). Notably, iso-piR-2158-L exerted a markedly stronger CSC-suppressive effect than iso-piR-2158-S (Figure 5D).

**Figure 6:**
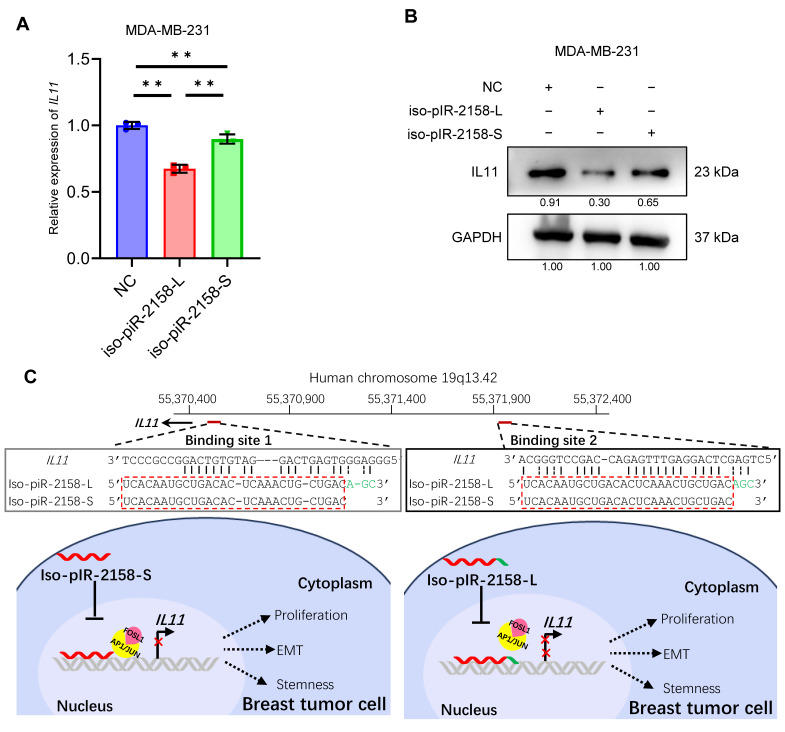
Distinct effects of iso-piR-2158-L and iso-piR-2158-S on suppression of IL11 signaling in MDA-MB-231 cells. (**A**,**B**) qRT-PCR and Western blot analyses showing that both piR-2158 isoforms suppress IL11 expression at both the mRNA (**A**) and protein (**B**) levels, with iso-piR-2158-L exerting a significantly stronger suppressive effect than iso-piR-2158-S. (**C**) Schematic illustrating the mechanism underlying the greater antitumor activity of iso-piR-2158-L than iso-piR-2158-S, driven by their distinct complementarity to the *IL11* promoter and resulting correspondingly distinct levels of IL11 suppression in breast cancer. The red boxes represent the consensus sequence shared by iso-piR-2158-L and iso-piR-2158-S, and the symbol “X” indicates transcriptional repression. Data are presented as mean ± SD (*n* = 3). ** *p* < 0.01. The uncropped blots are shown in Supplementary Materials.

**Distinct effects of iso-piR-2158-L and iso-piR-2158-S on suppression of IL11 signaling in breast cancer.** Our previous study has reported that piR-2158 acts as a transcriptional repressor of *IL11* by competing with the AP-1 transcription factor subunit FOSL1 for binding to the *IL11* promoter [17]. Herein, we assessed IL11 expression in MDA-MB-231 cells following transfection with either iso-piR-2158-L or iso-piR-2158-S. As a result, both piR-2158 isoforms suppressed IL11 at the levels of mRNA and protein, with iso-piR-2158-L exerting a significantly stronger inhibitory effect than iso-piR-2158-S (Figure 6A,B, Supplementary Table S3). Subsequently, we performed a sequence alignment analysis between the two piR-2158 isoforms and piR-2158-binding sites at the promoter region of *IL11*. As shown in Figure 6C, it is proposed that iso-piR-2158-L exhibits higher target-binding complementarity than iso-piR-2158-S, which may account for its greater antitumor activity across all assays tested.

## 4. Discussion

The malignant transformation of mammary epithelial cells (MECs) into invasive breast cancer cells is a multifactorial, multi-step process driven by diverse regulatory mechanisms, including genomic mutations, hormonal dysregulation, aberrant cellular signal transduction, tumor microenvironment remodeling, and immune evasion. Determining the molecular drivers of tumor initiation and progression will lead to the identification of novel therapeutic targets and development of innovative treatment strategies for breast cancer.

The role of small non-coding RNAs, particularly miRNAs, in regulating tumor initiation and progression has long been a core focus of cancer research. By contrast, the role of piRNAs in human cancer remains a formidable challenge to fully characterize, due to the unresolved uncertainties and inherent complexity of piRNA biogenesis, ribonucleoprotein complex assembly, and cancer cell-specific functions. Literature and our previous studies have demonstrated that some piRNAs play critical roles in cancer cells, most notably in cancer stem cells. For instance, piR-823 has been shown to induce ALDH-positive breast cancer stem cells [20]. piR-36712 has been shown to suppress breast cancer progression and chemoresistance by interaction with SEPW1 pseudogene SEPW1P [21]. One of our recent studies identified piR-2158 as a potential gene therapy target for breast cancer, as it inhibits mammary tumor stemness and tumor angiogenesis [17].

In this study, we identify a novel regulatory mechanism that modulates the antitumor function of piR-2158 in human breast cancer, which relies on post-transcriptional processing sites at the 3′ end of pre-piR-2158. We identified two types of piR-2158 isoforms in human MECs and breast cancer cells: a 31 nt long isoform (iso-piR-2158-L) and a 28 nt short isoform (iso-piR-2158-S). Gene expression analysis indicated that iso-piR-2158-L is dominantly expressed in normal MECs, while significantly downregulated in TNBC. Although both iso-piR-2158-L and iso-piR-2158-S exhibited tumor suppressive function in the TNBC cell lines we tested, iso-piR-2158-L exhibited stronger antitumor activity than iso-piR-2158-S. We propose that the downregulation of iso-piR-2158-L in cancer cells may contribute to tumorigenesis in TNBC, a hypothesis that requires further validation.

The mechanism underlying iso-piRNA biogenesis remains poorly characterized. Unlike iso-miRNAs, whose biogenesis has been frequently reported to arise from alternative Drosha processing and precise processing of the same primary transcript at multiple post-transcriptional steps, the regulatory mechanism of iso-piRNA biogenesis has not yet been elucidated. Multiple factors, including the Drosha and Dicer ribonucleases, RNA modifications, and RNA-binding proteins that recognize flanking sequences or structural elements of pre-miRNAs to promote alternative cleavage sites, are involved in regulating 5′or 3′end variation in iso-miRNAs. Aberrant iso-miRNA expression has been frequently reported in human diseases, including cancers [22,23,24,25,26,27]. Whether iso-piRNA biogenesis follows an analogous regulatory mechanism remains an open question.

Interleukin 11 (IL11) exerts potent oncogenic effects in breast cancer by driving tumor cell proliferation, epithelial-mesenchymal transition, cancer stemness, angiogenesis, and therapeutic resistance [28,29,30]. Our previous work demonstrated that piR-2158 binds the *IL11* promoter in a sequence-dependent manner, blocks the recruitment of the transcription factor FOSL1, and thereby inhibits *IL11* transcription and downstream IL11/STAT3 signaling [17]. In this study, our analyses indicate that iso-piR-2158-L more effectively suppresses IL11 expression than iso-piR-2158-S. Sequence alignment analysis reveals that the 3′end of iso-piR-2158-L exhibits greater base-pairing potential with the *IL11* promoter compared to iso-piR-2158-S. Based on these observations, we hypothesize that the differential antitumor effects of the piR-2158 isoforms are likely attributable to their distinct binding affinities for the *IL11* promoter—a mechanism requiring further validation through in vitro and in vivo experiments.

Several limitations remain in the current study. For instance, more accurate methods that enable direct discrimination of highly homologous small RNA isoforms, having distinct 3′ or 5′ ends with merely 1–3 nucleotide differences, need to be developed to double-check the current findings. The cohort of TNBC specimens and normal tissues included in this study is relatively small and restricted to only one breast cancer molecular subtype. The expression profiles and regulatory roles of these piRNA isoforms require further validation in larger-scale studies covering multiple molecular subtypes of breast cancer. For the mechanistic hypothesis that iso-piR-2158-L binds the *IL11* promoter with higher stability than iso-piR-2158-S, thereby exerting different antitumor effects against breast cancer, validation via in vitro and in vivo functional assays is still required. Although the current study performed gain-of-function assays to demonstrate that piR-2158 isoforms suppressed cell proliferation, migration, and cell stemness in MDA-MB-231 and Hs578T cells, complementary loss-of-function assays will strengthen the conclusion.

## 5. Conclusions

The current study identifies two novel piR-2158 isoforms in human breast tissue specimens that share an identical 5′ end sequence but have a distinct 3′ end: a 31 nt isoform (iso-piR-2158-L) and a 28 nt isoform (iso-piR-2158-S). Iso-piR-2158-L is enriched in normal mammary tissue, but downregulated in TNBC, where it functions as a tumor suppressor by inhibiting cancer cell proliferation, migration, and stemness. Additionally, we demonstrated that iso-piR-2158-L exerts stronger antitumor effects than iso-piR-2158-S, most likely due to the former having higher efficacy than the latter to suppress IL11 expression. Our findings not only reveal a piRNA isoform-based regulatory pathway in TNBC, but also highlight the functional relevance of piRNA post-transcriptional processing and tissue- or cell type-specific piRNA expression patterns.

## Data Availability

The original contributions presented in this study are included in the article/Supplementary Materials. Further inquiries can be directed to the corresponding authors.

## Supplementary Materials

The following supporting information can be downloaded at: https://www.mdpi.com/article/10.3390/cancers18142237/s1, Table S1: Primer sequences; Table S2: Sequences of piRNA mimics; Table S3: The band intensity values of Western blot in Figures.
