# Supplementary material for: Identification of Novel piR-2158 Isoforms and Their Distinct Antitumor Effects on Triple-Negative Breast Cancer
Source: Cancers (Basel). 2026 Jul 12;18(14):2237. doi: 10.3390/cancers18142237 (PMC13406357; doi:10.3390/cancers18142237)

Supplemental Figure

A

Original uncropped images for Figure 5C

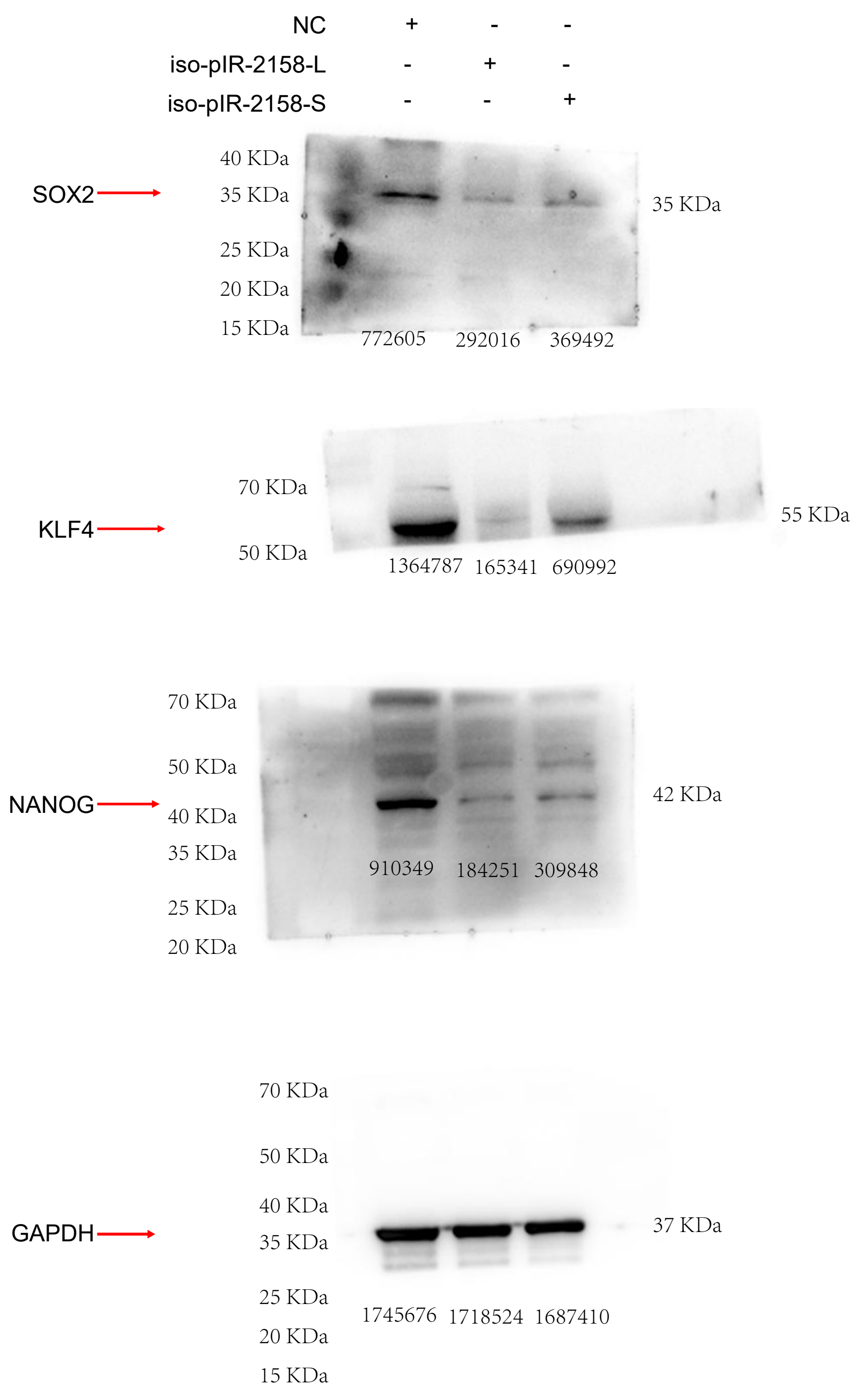

B

Original uncropped images for Figure 6B

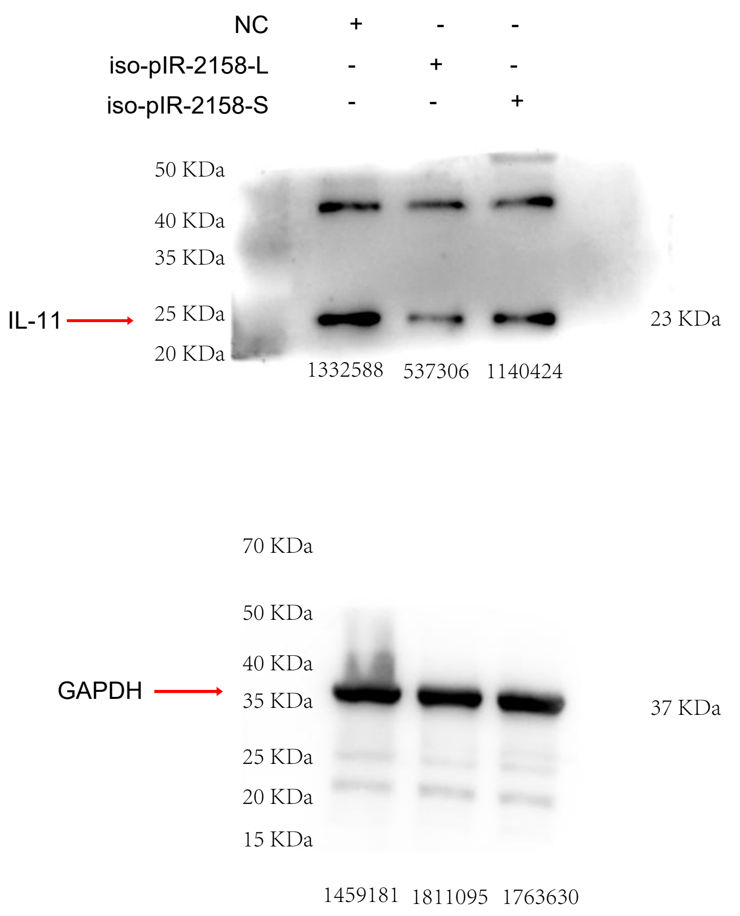

Supplement: Supplementary file 1 [file cancers-18-02237-s001.zip › Suplemental Figure.pdf]
